# Assessment of Knowledge About the Risk Factors of Chronic Liver Disease in Patients Admitted in Civil Hospital Karachi

**DOI:** 10.7759/cureus.5945

**Published:** 2019-10-20

**Authors:** Bushra Majid, Rimsha Khan, Zainab Junaid, Onaiza Khurshid, Syeda Hajrah Rehman, Syeda Naintara Jaffri, Batool Zaidi, Jabeen Zehra, Sabika Batool, Saman Altaf, Aliya Jatoi, Fatima Safina

**Affiliations:** 1 Internal Medicine, Dow University of Health Sciences, Karachi, PAK; 2 Obstetrics and Gynecology, Dow University of Health Sciences, Karachi, PAK

**Keywords:** chronic liver disease, hepatitis b, hepatitis c, cirrhosis, alcohol, awareness

## Abstract

Background

Chronic liver disease (CLD) encompasses a series of single or multifactorial insults to the liver, most common of which are hepatitis, alcohol abuse, and non-alcoholic liver disease. CLD represents a major public health problem worldwide as well as in Pakistan. Unfortunately, studies evaluating the awareness of its risk factors among people are quite scarce. The purpose of this study was to assess the knowledge about risk factors of CLD in patients admitted to Dr. Ruth K M Pfau, Civil Hospital Karachi (CHK).

Methods

This quantitative cross-sectional study was conducted at CHK, among 368 patients admitted to CHK, during the period from February 2018 to September 2018. Frequencies and percentages were evaluated for categorical variables using Statistical Package for Social Sciences (SPSS), version 22 (IBM Corp., Armonk, NY). Chi-square test was applied to determine if there was any significant association between the variables. A p-value of <0.05 was considered significant.

Results

A scoring scale was developed to assess the level of knowledge. We found that only 32% of our study population had good knowledge about the risk factors of CLD while majority of them (68%) had poor knowledge. Regarding individual risk factors, >60% patients were aware that obesity, high fat intake, prolonged use of drugs, use of alcohol and hepatitis B and C are risk factors of CLD, while most of them did not know diabetes to be a risk factor as well. 51.4% patients thought that hepatitis B and C could not be transmitted via sexual contact and from mother to her baby. There was a positive association between education level and awareness about the risk factors of CLD (p = 0.006).

Conclusion

Future interventions to increase public awareness about CLD should promptly be taken. The lack of knowledge about this disease is the main cause of its rapid increment and the reason why it is so prevalent in our part of the world especially Pakistan. More studies and nationwide awareness programs are needed to control its spread.

## Introduction

Chronic diseases are responsible for approximately 60% of deaths worldwide [1] among which chronic liver disease (CLD) accounts for almost 2 million deaths per year [2]. CLD is known as the stage when the regenerating power of the liver parenchyma is lost due to continuous injurious stimuli [3], resulting in liver failure which in turn due to its debilitating sequela, markedly decreases the quality of life, causing increased morbidity that ultimately ends up in premature death. CLD-related mortality figures in the USA have increased from being the tenth most leading cause of death in 2001 [4] to the ninth most common among males in 2016 [5]. In a developing country like Pakistan, incidence is much more common, being the fifth most common cause of death and the eleventh most common cause of disability [6].

Risk factors of CLD in Pakistan differ from those in rest of the world and include viral hepatitis (Hepatitis B and C), non-alcoholic steatohepatitis (NASH), diabetes mellitus, obesity and use of herbal and dietary supplements, while autoimmune hepatitis, Wilson disease, haemochromatosis and alcoholism are among the rare causes [7]. Among the mentioned risk factors, Pakistan has the second highest prevalence of hepatitis C in the world [8]. The traditional use of herbal medications also contributes to CLD, which is quite a norm in the country [9]. A survey conducted in Karachi in 2003, showed that 19.2% of the beds of medical wards of two major hospitals of the city, namely Civil Hospital Karachi (CHK) and Jinnah Post Graduate Medical Centre (JPMC) were occupied by CLD patients [10].

Pakistan, being a third world country, lacks resources to create awareness among people about the potential causes of CLD which has accentuated the burden of this disease manifold. Therefore, it is important to assess and evaluate the current knowledge of the people, specially when limited data exists due to lack of studies, particularly in Karachi. In our study, we aim to assess the knowledge and awareness of the patients admitted in CHK regarding the risk factors of CLD. The main reason for confining our study to only patients is that the admitted patients in hospitals have a greater risk of acquiring hepatitis C infection compared to the general population [11]. This study is essential to fill in the knowledge gap which can prove to be a great aid in planning future actions.

## Materials and methods

A cross-sectional study was conducted in CHK, during the period from February 2018 to September 2018. The sample size of 368 was calculated from OpenEpi.com using a confidence interval of 95%. Patients were selected via non-probability purposive sampling and all those who consented to respond were included in the study. All patients above 18 years of age, admitted to CHK, who understood Urdu or English were included in the study. Excluded from the study were patients less than 18 years of age and those who did not speak or understood English or Urdu. Patients who were mentally incapacitated due to any previous or current psychiatric illness were excluded from the study. Those who were unable to answer the questionnaire due to senile dementia or severe intercurrent disease were also excluded. No amputations were included in the study.

A structured questionnaire of 22 questions was used as the main study tool. The questionnaire was designed by the study team and tested via a pilot study. It was also translated to Urdu to remove any education bias. No question that would reveal the identity of the person was incorporated and confidentiality regarding personal information was maintained throughout. The questionnaire was divided into three sections.

(a) The first consisted of socio-demographic factors.

(b) The second part comprised of the factors that assessed the knowledge about the association of CLD with the risk factors such as diabetes mellitus, obesity, high fat intake, use of drugs/herbal medications, alcohol consumption, depression and chronic hepatitis B and C.

(c) The third part assessed the prevalence of screening for hepatitis B and C, the source of participants’ present knowledge and the willingness to acquire further information about CLD.

Data was collected from the study participants by medical students in person as an interview. An informed consent was obtained from all the participants verbally before interviewing them. Interviewers were well trained and a standard protocol was followed throughout to eliminate any chance of interviewer bias. Almost all the wards were covered during the data collection which included the following departments of ophthalmology, general surgery, general medicine, otorhinolaryngology, plastic surgery, orthopedics, cardiology, vascular surgery, neurosurgery and gynecology.

Data was analyzed using Statistical Package for the Social Sciences (SPSS) version 24.0 (IBM Corp., Armonk, NY). The descriptive statistical data was shown as frequency and percentage whereas chi-square was applied for comparative analysis. A p-value of <0.05 was considered significant. All the variables were computed and by which scoring was achieved.

## Results

Socio-demographic characteristics

A total number of 368 patients were studied. Majority (n = 157, 42.7%) were middle aged. Around half (n = 201, 54.6%) of the study participants were male. Nearly two-fifth of the patients had received no formal education (39.1%), whereas the percentage of people who received higher education was quite low, only 5.7% (n = 21) being graduates/post-graduates. About half of the study participants had a job (n = 198, 53.8%) while the rest were unemployed (n = 170, 46.2%) (Table 1).

**Table 1:** Demographics of included participants: (n = 368)

VARIABLES	FREQUENCY (n)	PERCENTAGE (%)
AGE (YEARS)		
18-30	127	34.5
30-50	157	42.7
>50	84	22.8
GENDER		
Male	201	54.6
Female	167	45.4
EDUCATION		
Uneducated	144	39.1
Primary	88	23.9
Secondary	115	31.3
Graduate/Post-graduate	21	5.7
WORKING STATUS		
Employed	198	53.8
Unemployed	170	46.2

Knowledge of respondents

Patients’ awareness about the risk factors were categorized among good and poor knowledge in accordance to their response which was scored and later assessed. A total score of 22 points was set among which patients scoring 13 points or below (<60%) were considered having a poor knowledge and those scoring above 13 points (≥60%) were regarded as having a good knowledge. Majority of the patients had a good knowledge about the association of risk factors such as obesity, high fat intake, prolonged use of drugs, use of alcohol and Hepatitis B and C with CLD. Patients had moderate knowledge regarding risk factors like prolonged use of herbal drugs and prevention of Hepatitis B via vaccine. Poor knowledge was observed with diabetes and depression as risk factors for CLD and for the transmission of Hepatitis B and C via physical contact, dirty food or water and prevention of Hepatitis C via vaccine. The response for knowledge is summarized in Table 2.

**Table 2:** Awareness of participants towards risk factors of CLD: (n = 368) CLD: Chronic liver disease

RISK FACTORS ASSESSED	CORRECT, n (%)	INCORRECT, n (%)
Diabetes mellitus as a risk of CLD	137 (37.2)	231 (62.8)
Sugar control and risk of CLD	124 (33.7)	244 (66.3)
Obesity as a risk of CLD	206 (56)	162 (44)
High fat intake as a risk of CLD	269 (73.1)	99 (26.9)
Prolonged use of drugs as a risk of CLD	217 (59)	151 (41)
Prolonged use of herbal medications as a risk of CLD	150 (40.8)	218 (59.2)
Alcohol as a risk of CLD	296 (80.4)	72 (19.6)
Types of alcohol and risk of CLD	153 (41.6)	215 (58.4)
Amount of alcohol consumed and risk of CLD	214 (58.2)	154 (41.8)
Duration of alcohol consumption and risk of CLD	216 (58.7)	152 (41.3)
Hepatitis B and C as risk factors for CLD	260 (70.7)	108 (29.3)
Transmission via non-screened blood transfusion	219 (59.5)	149 (40.5)
Transmission via unhygienic dental check up	188 (51.1)	180 (48.9)
Transmission via dirty food and water intake	20 (5.4)	348 (94.6)
Transmission via physical contact (hand shake, touching) with an infected person	112 (30.4)	256 (69.6)
Transmission via infected mother to her fetus	179 (48.6)	189 (51.4)
Transmission via sharing razors, needles and syringes of an infected person	231 (62.8)	137 (37.2)
Transmission via sexual contact with an infected person	179 (48.6)	189 (51.4)
Transmission via sharing tooth brush, glass and cups with an infected person	190 (51.6)	178 (48.4)
Prevention of Hepatitis B via a vaccine	195 (53)	173 (47)
Prevention of Hepatitis C via a vaccine	0	368 (100)
Depression as a risk for CLD	0	368 (100)

Level of awareness

**Figure 1:**
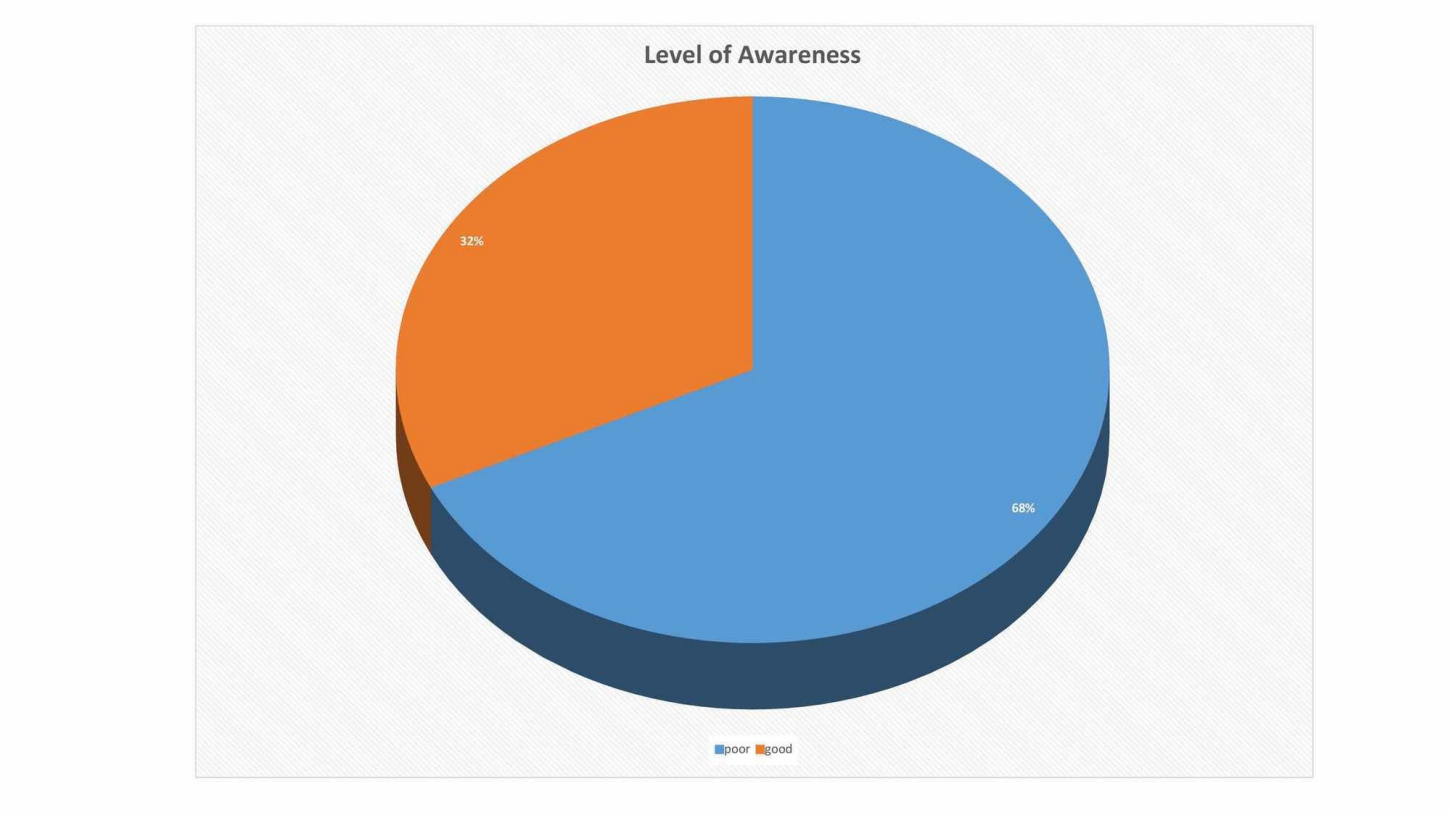
Level of awareness

More than half (n = 250, 68%) of the patients had poor knowledge while 32% (n = 118) had good knowledge regarding risk factors of CLD as depicted in Figure 1.

Willingness of patients to know about CLD and its risk factors

Willingness of patients to learn about CLD and its risk factors is shown in Table 3.

**Table 3:** Willingness to know about CLD CLD: Chronic liver disease

WILLINGNESS OF PATIENTS	FREQUENCY (n)	PERCENTAGE (%)
Yes	300	81.5
No	68	18.5
Total	368	100

Prevalence of screening for hepatitis B and C

Nearly more than 3/5th of the participants (n = 229, 62.2%) had never been screened for hepatitis B and C, whereas the rest (n = 139, 37.8%) claimed that they had undergone a prior screening test (Table 4).

**Table 4:** Screening for hepatitis B and C

PRIOR SCREENING	FREQUENCY (n)	PERCENTAGE (%)
Yes	139	37.8
No	229	62.2
Total	368	100

Source of knowledge of patients

**Figure 2:**
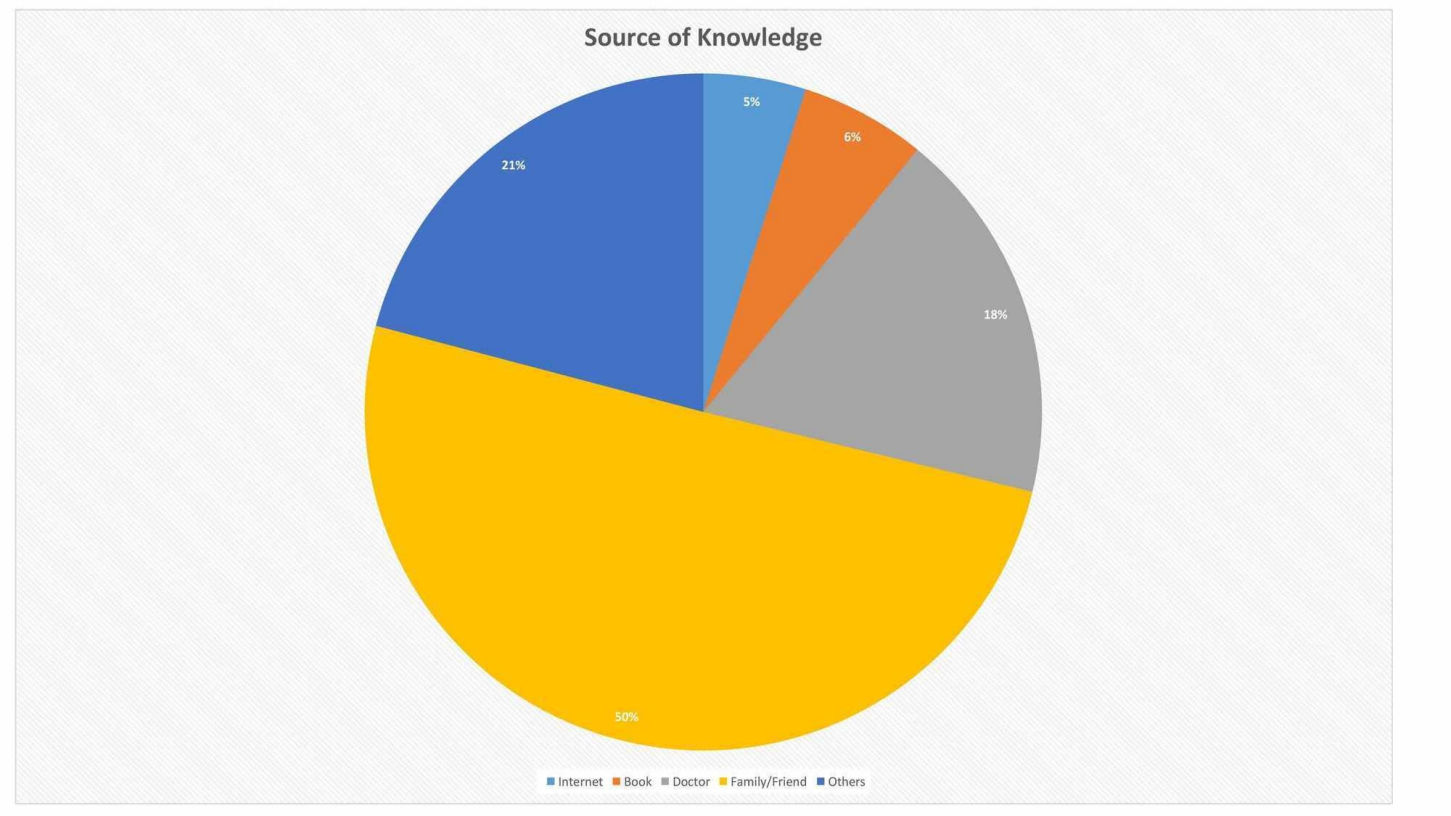
Source of knowledge

The main source of knowledge about the risk factors of CLD (n = 185, 50.3%) was family and friends, while books and internet comprised of only (n = 22, 6%) and (n = 18, 4.9%) respectively as shown in Figure 2.

Association of subject’s knowledge with socio-demographic variables

There was a significant association between high level of education and good awareness of the risk factors of CLD (p = 0.006) as depicted in the table below (Table 5).

**Table 5:** Association between level of knowledge and demographics of the participants

DEMOGRAPHICS	GOOD KNOWLEDGE (118) n (%)	POOR KNOWLEDGE (250) n (%)	p-value
Age (years)			
18-30	39 (33.05)	88 (35.2)	0.910
31-50	52 (44.06)	105 (42)	
Above 50	27 (22.88)	57 (22.8)	
Gender			
Male	66 (55.9)	135 (54)	0.728
Female	52 (44.06)	115 (46)	
Education			
Uneducated	38 (32.2)	106 (42.4)	0.006
Primary	36 (30.5)	52 (20.8)	
Secondary	32 (27.11)	83 (33.2)	
Graduate/Post Graduate	12 (10.16)	9 (3.6)	
Occupation			
Unemployed	52 (44.06)	118 (47.2)	0.574
Employed	66 (55.93)	132 (52.8)	

## Discussion

In our study, we aimed to assess the knowledge of patients admitted to CHK, about the risk factors of CLD. CLD represents a major global health problem but unfortunately, the studies regarding the assessment of its awareness are very limited. The findings of our study suggested that the awareness about the risk factors of chronic liver diseases is alarmingly low in our population. Although, the knowledge about different risk factors varied, overall, only 32% of the respondents had adequate knowledge. The remaining 68%, which comprised the majority, had poor knowledge.

In a study conducted in Saudi Arabia regarding liver cirrhosis, which is a part of CLD, 54% of Saudi population had good knowledge while 46% had poor knowledge [12]. The most probable reason for majority of their population having good knowledge is the higher literacy rate as compared with that of Pakistan, which supports our results, i.e., majority of the people having poor knowledge. The findings of our study suggested that majority of the uneducated people had poor knowledge while most graduates had better knowledge. This indicates that there was significant association of education level and awareness level as represented by p value of 0.006. However, these are contrary to the results of a study conducted in Aga Khan University Hospital on the assessment of knowledge of hepatitis B and C in which the study participants, despite being educated had poor knowledge of these diseases [13]. Association between knowledge and other demographics was not found to be significant.

In the recent times, there has been a surge in alcohol abuse in Pakistan resulting in many of its complications as well [14]. So, it is not surprising that nearly 80% of the respondents knew alcohol to be a risk factor of liver diseases. The risk of developing liver damage greatly depends upon the amount of alcohol consumed. The minimum alcohol intake associated with a significant increase in risk, ranges from 40-80 gm/day [15]. This fact was also well known by more than half of the respondents (58.2%). Similarly, the type of alcohol consumed may also influence the risk of developing CLD. In a survey conducted in Denmark, certain types of alcohol were more likely to be associated with liver diseases as compared to others but our study found that only 41.6% of the respondents were familiar with this knowledge [16].

Obesity and high fat intake are significant risk factors of chronic liver diseases. 56% of the people considered obesity as a risk factor of CLD. However, most of them, i.e., 73% were convinced that high fat intake can also lead to liver damage. A previous study by Said et al. reported similar results with 60% of respondents giving the correct response [17].

Long-term use of certain hepatotoxic drugs like anti-tuberculous drugs (ATD), acetaminophen, certain antiretroviral, protease inhibitors and many others can lead to CLD [18]. More than half of the respondents (59%) were aware of this risk factor.

In our study, we found that most of the people had poor knowledge regarding diabetes as risk factor of CLD despite its very high prevalence in Pakistan, with almost 9.8% of the population suffering from it [19]. Similar responses were found regarding herbal medications and more than half of the respondents were not aware that a number of herbal medications can cause damage to liver, as also depicted by a study conducted in Thailand [20]. According to WHO, approximately 80% of world’s population of developing countries still depends on alternative systems of medicine including herbal medicines [21]. When the respondents were asked if they considered depression as a risk factor of CLD, interestingly, majority of them replied in the affirmative. Nevertheless, a study on the prevalence of depression in patients with CLD summarized that patients afflicted with CLD manifested different degrees of depressive symptoms but there are no studies that show depression to be a risk factor of CLD [22]. This points out towards the misconception that people have regarding depression.

In Pakistan, hepatitis C remains the most common antecedent of CLD [8]. Having adequate knowledge about this potentially preventable illness can, in the long run, drastically reduce the burden of CLD, especially in developing countries. Hospital-acquired hepatitis C infection is very common among patients. In a review by Pozzetto et al., it was highlighted that patients admitted to hospital are particularly at high risk for acquiring hepatitis C infection compared to general population [11], so we focused more upon hepatitis B and C during our survey. Upon assessing the level of awareness of hepatitis B and C as risk factors for CLD, majority (70%) gave a correct response. Despite having a good knowledge about hepatitis B and C as risk factors of CLD, very few of them knew about the correct modes of their transmission. These results are comparable to the study conducted in a hospital in Sindh in 2015, which showed that majority of their population had misconceptions or had poor knowledge about hepatitis C transmission [23]. The results of our study showed that about 94.6% people wrongly believed that “transmission via dirty water and food intake” is possible and 69.6% people had misconception regarding transmission of these viruses through physical contact like hand shaking, hugging, kissing, etc. with an infected person. These findings were opposed by the results of a study conducted among non-medical students of universities in Karachi where nearly 70% of the respondents had the correct belief that hepatitis B and C virus (HBV and HCV) were not spread through contaminated food and water [24]. Only half of the respondents answered correctly about transmission via transfusion of non-screened blood, unhygienic dental check-up, unprotected sexual contact, sharing of razors, needles, syringes, and via mother to fetus [25]. Half of the people correctly knew that hepatitis B is a vaccine preventable disease but astonishingly, most of the people had a great misconception that hepatitis C could also be prevented by vaccine. These findings are supported by the results of a study conducted in Aga Khan University Hospital which showed that nearly 66% of the people had the same misconception about hepatitis C vaccine [13].

When the respondents were asked if they had ever gotten themselves screened for hepatitis B and C, most of them (62.2%) denied being tested and thus were unaware of their infectious or carrier status. Only 37.8% participants had been screened. This finding is quite alarming as according to a survey conducted by Pakistan Medical Research Council (PMRC), it is estimated that in Pakistan, about 12 million individuals are affected by hepatitis B and C. This comprises of 7.4% of the population and out of this, 2.4% are infected with hepatitis B and 4.9% are infected with hepatitis C [26]. The disparities in screening for HBV and HCV can be attributed to poverty, lack of awareness, language barriers, and difficulty in accessing healthcare.

Upon assessing the source of knowledge of the respondents, we observed that the main sources were “family and friends” (50%). However, this is not a very authentic source as many myths and misconceptions circulate among the common people. Books (6%) and internet (5%) were not common sources of knowledge as most of our respondents were uneducated.

Regarding the respondents’ interest in gaining knowledge about the CLD, surprisingly, we found that nearly 80% of them were willing to know more about the risk factors of these diseases. This suggests that more and more health awareness programs should be carried out to educate people as ignorance can potentially lead to an inevitable state of disease progression.

## Conclusions

Due to lack of education and awareness, CLD is rapidly spreading and becoming a major health burden due to its long-term effects. Currently, knowledge propagation by means of newspaper, pamphlets, banners, and television is not utilized effectively, either because of limitations due to language differences throughout the country, affordability, or illiteracy among the general public. Hence, there is an immediate need to conduct nationwide awareness programs through all possible mediums to raise the awareness of CLD in the Pakistani population, where the disease continues to afflict several each year. This is one effective way to combat this endemic disease.
